# Aberrant patterns of neural activity when perceiving emotion from biological motion in schizophrenia

**DOI:** 10.1016/j.nicl.2018.08.014

**Published:** 2018-08-09

**Authors:** Amy M. Jimenez, Junghee Lee, Eric A. Reavis, Jonathan K. Wynn, Michael F. Green

**Affiliations:** a Desert Pacific MIRECC, VA Greater Los Angeles Healthcare System, 11301 Wilshire Blvd., Los Angeles, CA 90073, USA; b Department of Psychiatry and Biobehavioral Sciences, University of California, Los Angeles, 405 Hilgard Ave., Los Angeles, CA 90095, USA

**Keywords:** pSTS, posterior superior temporal sulcus

## Abstract

Social perceptual deficits in schizophrenia are well established. Recent work suggests that the ability to extract social information from bodily cues is reduced in patients. However, little is known about the neurobiological mechanisms underlying this deficit. In the current study, 20 schizophrenia patients and 16 controls completed two tasks using point-light animations during fMRI: a basic biological motion task and an emotion in biological motion task. The basic biological motion task was used to localize activity in posterior superior temporal sulcus (pSTS), a critical region for biological motion perception. During the emotion in biological motion task, participants viewed brief videos depicting happiness, fear, anger, or neutral emotions and were asked to decide which emotion was portrayed. Activity in pSTS and amygdala was interrogated during this task. Results indicated that patients showed overall reduced activation compared to controls in pSTS and at a trend level in amygdala across emotions, despite similar task performance. Further, a functional connectivity analysis revealed that controls, but not patients, showed significant positive connectivity between pSTS and left frontal regions as well as bilateral angular gyrus during the emotion in biological motion task. These findings indicate that schizophrenia patients show aberrant neural activity and functional connectivity when extracting complex social information from simple motion stimuli, which may contribute to social perception deficits in this disorder.

## Highlights

• Perception of social information from bodily cues is impaired in schizophrenia.
• We examined neural correlates of perception of emotion from biological motion.
• Activity in amygdala and posterior superior temporal sulcus was reduced in patients.
• pSTS functional connectivity with frontal and parietal regions was reduced in patients.
• Aberrant neural responses may contribute to social perceptual deficits in schizophrenia.

## 1 Introduction

We live in a dynamic visual world. The detection of motion and the ability to obtain information from motion is an important visual perception ability (Blakemore and Decety, 2001; Knoblich and Flach, 2001). Reflecting that importance, humans are remarkably adept at recognizing coherent motion (Mingolla et al., 1992; Williams and Sekuler, 1984), and distinguishing actions performed by other humans from non-human movement (Ahlström et al., 1997). Human movement is considered to be a type of biological motion. Humans can recognize biological motion even when the patterns of movements are portrayed by nothing more than a handful of light points attached to the head and major joints of the body, as in point-light animations (e.g., (Blake and Shiffrar, 2007; Grossman et al., 2000; Johansson, 1973).

Thus, biological motion is an important aspect of social cue perception. From observing biological motion (e.g., gestures and gait) we gather socially relevant information and make inferences about the intentions and motivations of others. We can also infer emotion based on particular patterns of human movement displayed by point-light animations (e.g., (Atkinson et al., 2004; Heberlein et al., 2004).

In healthy controls, there is robust evidence that activity in the posterior portion of the superior temporal sulcus (pSTS) is critical for biological motion perception (Allison et al., 2000). Activity in pSTS is associated with perception of static images of the face and body as well as movements of the eyes, mouth, hands, and body (Beauchamp et al., 2003; Puce and Perrett, 2003). More broadly, the pSTS is part of a large neural circuit involved in the perception of intention from action and response to objects and events of social and emotional relevance (Blake and Shiffrar, 2007; Zacks et al., 2001). Primate studies, as well as more recent neuroimaging studies in humans, have identified other key regions within this circuit, including amygdala and orbitofrontal cortex, which receive projections from pSTS (Aggleton et al., 1980; Bonda et al., 1996; de Gelder, 2006; Ethofer et al., 2011). The amygdala, in particular, is responsive to emotional facial expressions and complex body movements (e.g., (Adolphs et al., 1999; Conty et al., 2012; Wang et al., 2014) and has been shown to modulate activity in STS neurons (Aggleton et al., 1980; Blake and Shiffrar, 2007). That modulation has been shown to vary as a function of the emotional content of an action (e.g., (Labar et al., 2003; Wheaton et al., 2001).

In schizophrenia, the ability to extract social information, including emotion, from bodily cues is reduced (e.g., (Kern et al., 2013; Kim et al., 2005; Okruszek et al., 2015; Peterman et al., 2014; Strauss et al., 2015; Vaskinn et al., 2016). A recent meta-analysis of 6 published studies found an mean effect size for reduced detection of emotion in biological motion of −0.61 (Okruszek and Pilecka, 2017). However, little is known about the neurobiological mechanisms underlying deficits in biological motion detection or the ability to extract more complex information such as emotion from biological motion in schizophrenia.

There have been only a few studies to examine the structural or functional neuroanatomy of impaired basic biological motion perception using point-light animations in schizophrenia. Kim et al. (Kim et al., 2011), in a series of experiments, used two sets of basic biological motion fMRI tasks – localizer tasks and the primary experimental paradigm. The pSTS localizer task was a simple block-design with alternating blocks of biological and scrambled motion designed to identify BOLD activity in pSTS. The experimental paradigm was a more complex event-related task which required participants to judge whether a given motion stimulus (biological, scrambled, and 37% scrambled) depicted a human activity or not. For the pSTS localizer task, patients activated pSTS to the same extent as controls. This finding was replicated by Hashimoto et al. (Hashimoto et al., 2014) in a separate study using a similar task. However, during the more complex discrimination task, Kim et al. found that patients with schizophrenia showed an abnormal pattern of pSTS activity compared to controls. Specifically, pSTS activation was selective to biological versus non-biological (i.e., scrambled) motion in controls, but not in patients. Finally, Matsumoto et al. (Matsumoto et al., 2017) examined the relationship between behavioral performance on a basic biological motion perception task and regional gray matter volume. They found that task accuracy was positively correlated with gray matter reductions in the middle and anterior portion of right STS in schizophrenia. No studies to date have examined the neural basis of detection of emotion in biological motion.

To address this gap in this literature, the current study utilized two fMRI tasks using point-light animations in controls and patients with schizophrenia. First, a basic biological motion task was used as a localizer task to activate pSTS. This task has reliably activated pSTS (Hashimoto et al., 2014) and has been used previously as a pSTS localizer (Kim et al., 2011). We hypothesized that patients would activate pSTS to the same extent as controls during this task. Second, an emotion in biological motion task was used to examine neural activation associated with emotion judgments from biological motion in two ROIs: pSTS and amygdala. We also examined functional connectivity between pSTS and amygdala, as well as between pSTS and the rest of the brain, during emotion judgments. We chose the pSTS as the seed region for the connectivity analyses given its central role in the neural network associated with perception of human biological motion (e.g., (Kim et al., 2011)). We hypothesized that during the emotion in biological motion task patients would show reduced activation in pSTS and amygdala, and that functional connectivity associated with the task would be reduced in patients.

## 2 Methods

### 2.1 Participants

Twenty patients with schizophrenia (7 female) and 16 healthy controls (5 female) completed the study. Patients were recruited from community outpatient treatment clinics and met diagnostic criteria for schizophrenia based on the Structured Clinical Interview for DSM-IV Axis I Disorders (SCID) (First et al., 1997b). Controls were recruited through internet postings and interviewed with the SCID-I and portions of the Structured Clinical Interview for DSM-IV Axis II Disorders (SCID-II) (First et al., 1997a).

All participants were between 18 and 65 years of age, and were excluded from participation if they had: an identifiable neurological disorder, history of loss of consciousness for more than 1 h, or were not sufficiently fluent in English to consent and understand procedures. Additional exclusion criteria for patients was self-reported substance abuse in the past month or dependence in the last six months, and indication of IQ < 70 based on chart review. Additional exclusion criteria for controls was a first-degree relative with schizophrenia or another psychotic disorder, a personal history of schizophrenia or other psychotic disorder, bipolar disorder, or recurrent depression, a lifetime history of substance dependence or any substance abuse in the last 6 months, and any of the following Axis II disorders: avoidant, paranoid, schizoid, or schizotypal.

### 2.2 Clinical measures

Psychiatric symptoms in patients were evaluated using the 24-item version of the Brief Psychiatric Rating Scale (BPRS) (Ventura et al., 1995) and Scale for the Assessment of Negative Symptoms (SANS) (Andreasen, 1982). For the BPRS we report the total score and the mean for the positive symptom factor (Kopelowicz et al., 2008). For the SANS we report the sum of four global scales: Affective Flattening, Alogia, Avolition-Apathy, and Anhedonia-Asociality. Functional outcome was assessed with the Role Functioning Scale (RFS) (Goodman et al., 1993; McPheeters, 1984).

All clinical assessments were conducted by interviewers trained to reliability through the Treatment Unit of the Department of Veterans Affairs VISN 22 Mental Illness Research, Education, and Clinical Center (MIRECC) based on established procedures (Ventura et al., 1998). The study protocol was reviewed and approved by the Institutional Review Board of the University of California, Los Angeles. All participants provided written informed consent after procedures were fully explained.

### 2.3 Procedures

#### 2.3.1 Activation paradigms

**Fig. 1:**
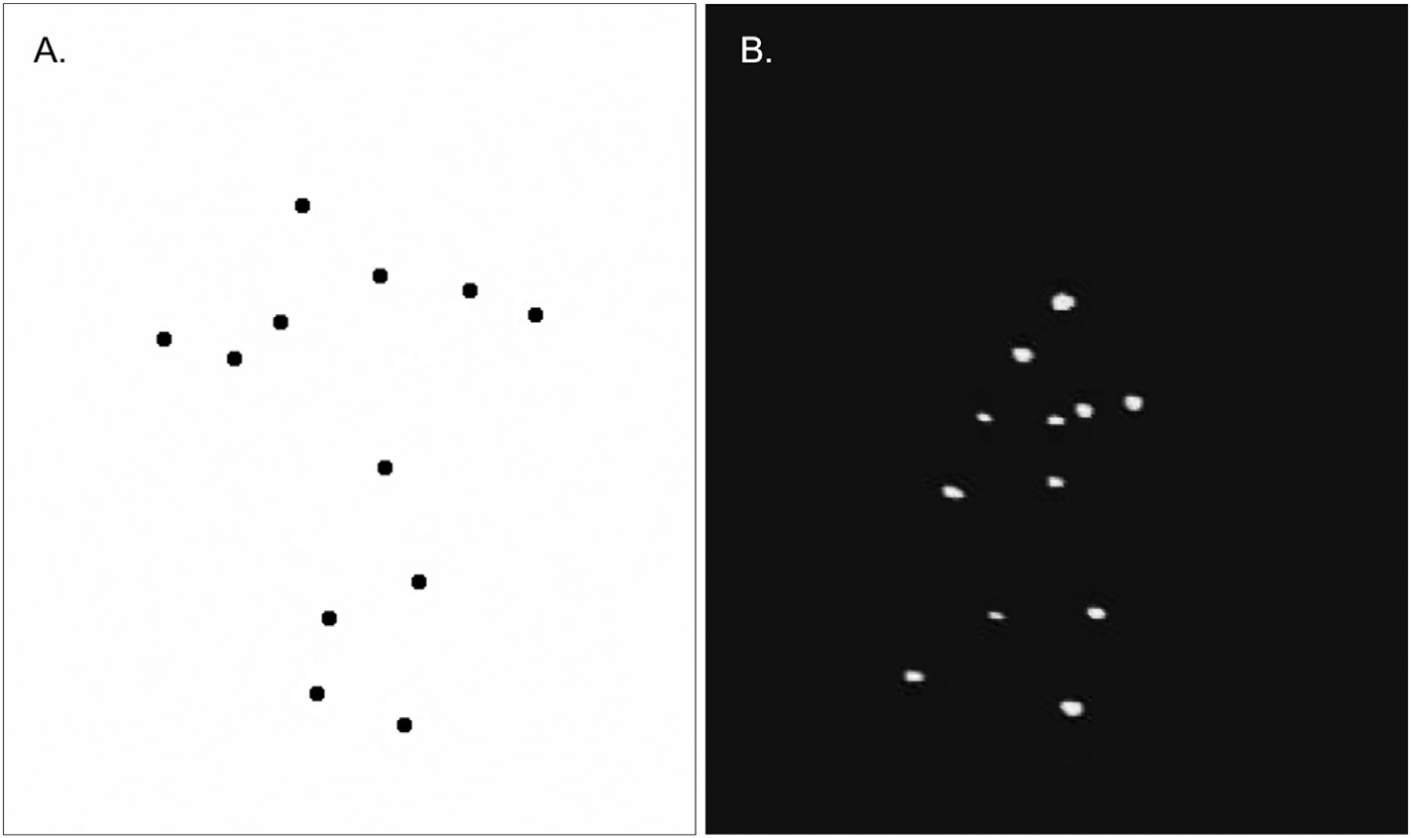
Example point-light animation stimuli from A.) Basic Biological Motion Task and B.) Emotion in Biological Motion Task.

Participants completed two tasks involving point-light animations during fMRI (Fig. 1). The first was the Basic Biological Motion Task (Jahshan et al., 2014; Kim et al., 2011). The second task was the Emotion in Biological Motion Task (Heberlein et al., 2004; Kern et al., 2013). The tasks were generated using *E*-Prime version 2.0 professional (Psychology Software Tools, USA). Prior to the scan session, participants completed practice trials to become familiar with each task.

Basic biological motion task (localizer): Stimuli consisted of 1 s animations of point-light walkers; each animation video was comprised of 12 black dots presented on a white background at central fixation. The dots were arranged and animated in a manner that corresponded to human (e.g., walking, jumping) or random movement (Fig. 1A). Participants viewed 12 alternating blocks of human (0% scrambled) and random (100% scrambled) movement. Each block included 7 animations with an inter-stimulus interval (ISI) of 1 s. A 1 s fixation cross was presented during each ISI. A 4 s fixation cross was presented between each block. To maintain the participant's attention, each block required performance of a 1-back task in which they were required to press a button whenever the current animation was identical to the one appearing immediately before it. Participants had 2 s to make their response; they could respond at any time after stimulus onset and prior to the onset of the next video.

Emotion in biological motion task (Emo Bio): Stimuli consisted of 20 animations of point-light walkers (average duration: 8.05 s, standard deviation: 6.7 s); videos were comprised of 12 white dots presented on a black background at central fixation. The dots were arranged and animated in a manner that depicted happiness, fear, anger, or neutral human emotion (Heberlein et al., 2004; Kern et al., 2013). A 1 s fixation cross was presented prior to each animation. After stimulus offset, the screen prompted the participant to make their response. Participants were asked to decide which emotion was being portrayed by pressing a corresponding button on a 4-button response box. Participants had 4 s to make their response. Responses triggered progression to a blank screen for the remainder of the 4 s response period. Each animation was followed by a “null” trial consisting of a fixation cross (mean duration: 4.25 s, range: 2.5–7.5 s).

#### 2.3.2 fMRI data acquisition and processing

All scanning was performed using two identical 3 T Siemens Tim Trio scanners running identical software at the UCLA Ahmanson-Lovelace Brain Mapping Center and the UCLA Staglin Center for Cognitive Neuroscience. Each scanner utilized the same quality assurance protocols daily to assess scanner performance. There was no difference between scanners in terms of number of subjects per group or signal-to-noise ratio (Supplementary Table 1).

Functional runs were acquired with T2*-weighted blood oxygen level-dependent (BOLD) gradient echo planar imaging (EPI) sequences for each activation task (TR = 2500 ms, TE = 35 ms, flip angle = 75°, 38 interleaved slices, slice thickness = 3.3 mm; matrix = 64 × 64; FOV = 192 mm; in-plane resolution = 3x3mm). Two sets of structural images were acquired from each subject for anatomical localization and registration of functional data: a high-resolution T1-weighted magnetization prepared rapid-acquisition gradient echo image (MPRAGE) [TR = 1900 ms; TE = 3.43 ms; flip angle = 9°; 160 sagittal slices; slice thickness = 1 mm; matrix = 256 × 256; FOV = 256 mm; voxel size = 1x1x1mm] and a T2-weighted matched-bandwidth high-resolution scan with the same slice prescription as the EPI [TR = 6000 ms; TE = 66 ms; flip angle = 90°; 38 slices; slice thickness = 3.3 mm; matrix = 128 × 128; FOV = 192 mm; voxel size = 1.5 × 1.5 × 3.3 mm].

Image preprocessing was carried out using the FMRIB Software Library (FSL v5.0.9; Analysis Group, Oxford, UK). Preprocessing steps included high-pass filtering (100 s cut-off), spatial smoothing (5 mm FWHM), skull stripping (Smith, 2002), and registration. Registration was carried out using FSL's FLIRT (FMRIB's Linear Image Registration Tool v6.0) (Jenkinson and Smith, 2001). Each run of individual EPI data was registered first to the co-planar matched-bandwidth T2-weighted image (affine transformation; 6 degrees of freedom), then to the T1-weighted MPRAGE (Boundary-Based Registration, BBR) (Greve and Fischl, 2009) and finally, to Montreal Neurological Institute (MNI) standard space (affine transformation, 12 degrees of freedom).

We addressed potential motion artifacts in several ways. Motion estimates were inspected to ensure that relative mean displacement did not exceed 0.5 mm for any participant. In addition, all images were realigned to the middle volume using MCFLIRT and movement parameters calculated by MCFLIRT were modeled as nuisance covariates (Jenkinson et al., 2002; Jenkinson and Smith, 2001). Finally, the FSL motion outliers tool was used to remove the effects of any time-points corrupted by motion beyond what realignment and linear motion parameter regression methods can fix.

#### 2.3.3 Behavioral analyses

For the demographic data, independent samples *t*-tests and chi-square tests were used to assess group differences for continuous and categorical variables, respectively. For the localizer task, behavioral data were collected to ensure participants were attending to the task but these data were not analyzed as a primary outcome variable. For the Emo Bio task, separate independent samples *t*-tests were used to assess group differences in response time and accuracy across condition (emotion type). Analyses measured significance at the 0.05 level, two-tailed. Given our relatively small sample size, we also give weight to any findings with an effect size of medium or greater.

#### 2.3.4 fMRI analyses

Analysis of fMRI data was performed separately for the two tasks. For both tasks, we used a general linear model (GLM) approach with FEAT (FMRI Expert Analysis Tool v6.0), and a timing model based on a double-gamma hemodynamic response function. In the first-level analysis, linear contrasts of trials by condition were created for each subject. The primary contrast of interest for the Basic Biological Motion Task was “biological” > “non-biological.” For the Emotion in Biological Motion Task, the primary contrasts of interest were each condition (“happy,” “afraid,” “angry,” “neutral”) versus baseline.

**Fig. 2:**
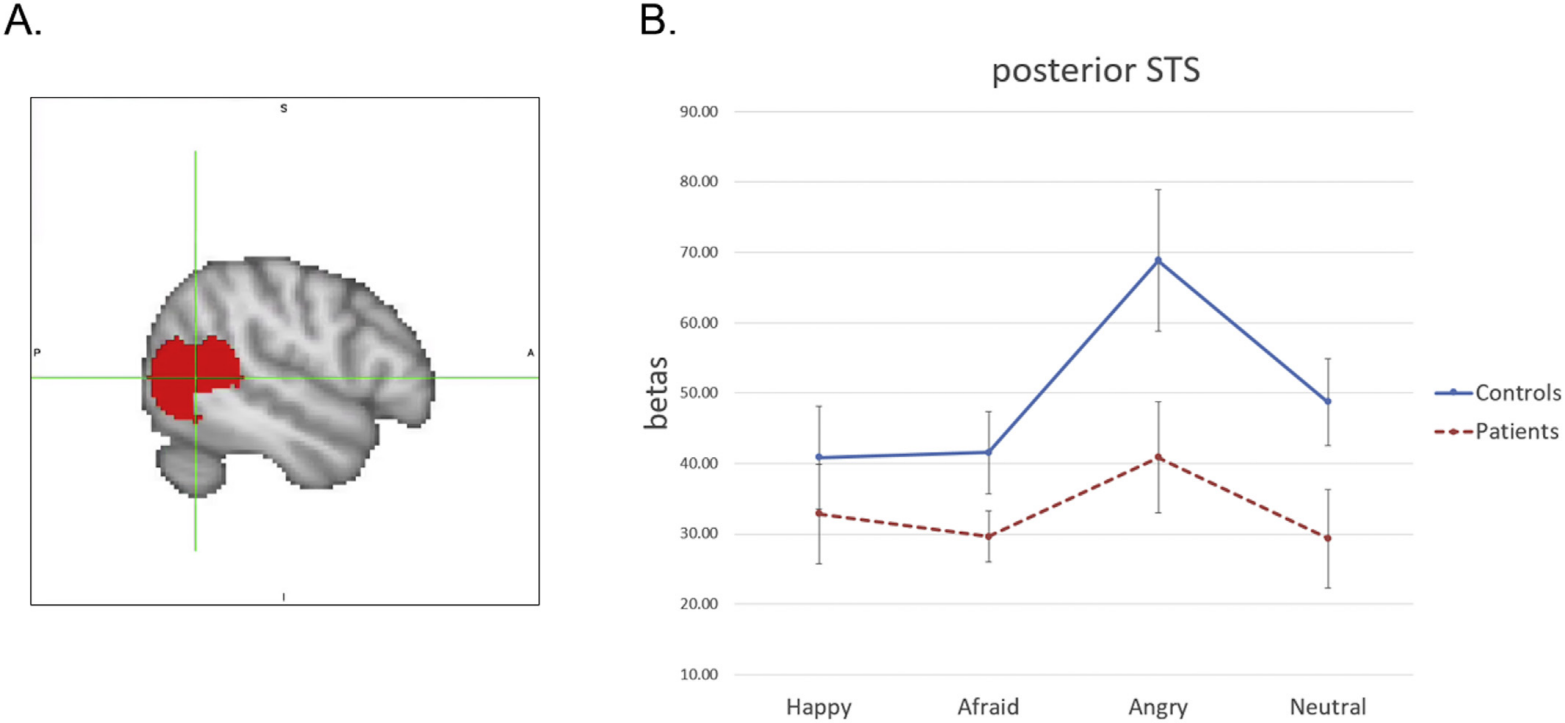
pSTS ROI - emotion in biological motion data. A. Location of ROI; crosshairs centered on the following coordinates (x, y, z) in MNI standard space: 52, −56, 6. B. fMRI activity in pSTS for each group and each condition (emotion type). Controls showed significantly greater activation than patients across conditions. Across groups, there was significantly greater activation for the anger condition compared to the fear and neutral conditions. Blue solid = controls; Red dash = patients. Bars represent plus or minus the standard error of the mean. Abbreviations: pSTS, posterior superior temporal sulcus; ROI, region-of-interest; MNI, Montreal Neurological Institute. (For interpretation of the references to color in this figure legend, the reader is referred to the web version of this article.)

Localizer task: The primary goal of the localizer task analyses was to identify voxels responsive to biological motion within bilateral pSTS. An anatomically constrained functional region of interest (ROI) for pSTS was created using a two-step procedure (Kim et al., 2011). First, a 20 mm diameter spheres were created around peak coordinates in MNI standard space (x = ±52, y = −56, z = 6) identified by a recent meta-analysis (Van Overwalle, 2009). Next, contiguous voxels within that anatomical region of cortex were identified that were significantly activated at the group level (all subjects combined) by biological motion relative to scrambled motion at a false discovery rate (FDR) of q < 0.05 (Fig. 2A).

**Fig. 3:**
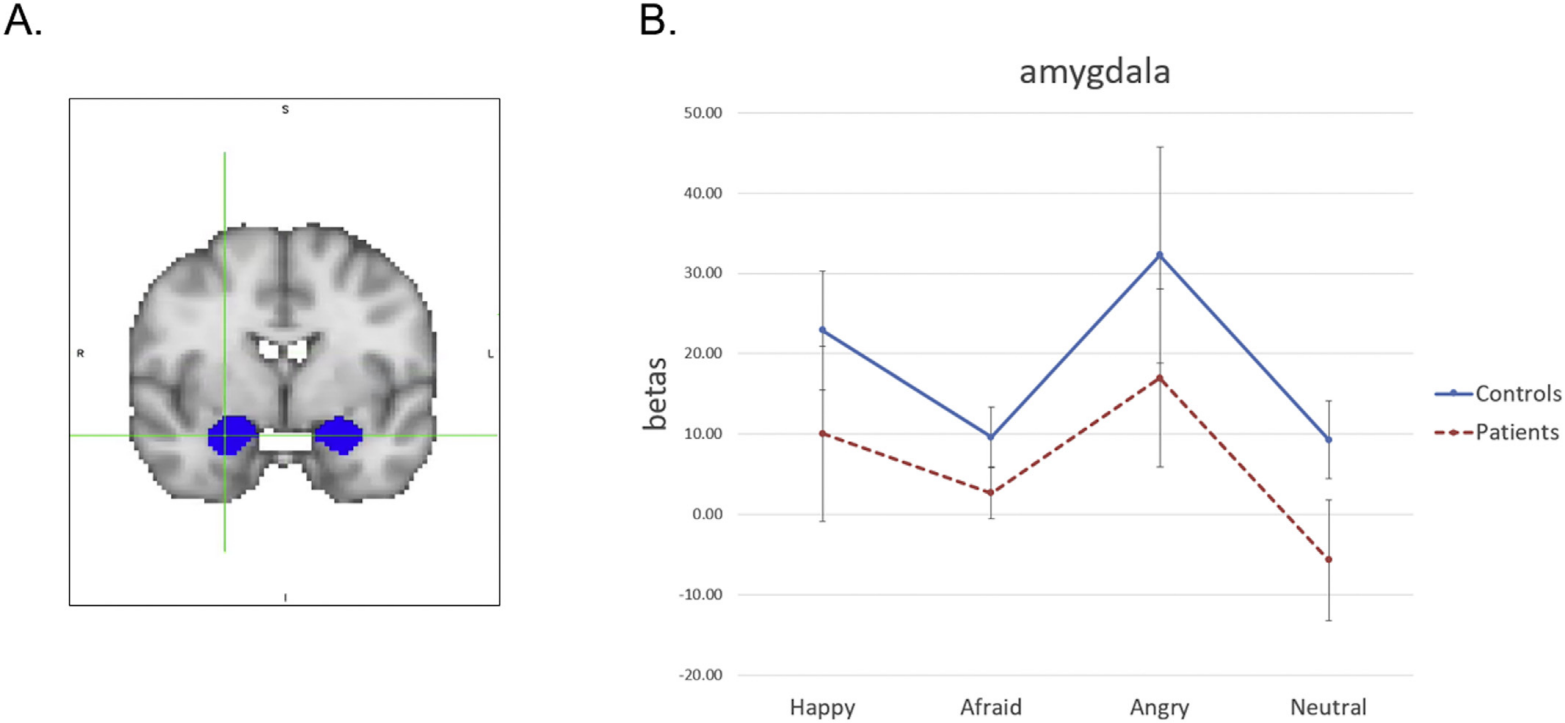
Amygdala ROI – emotion in biological motion data. A. Location of ROI; crosshairs centered on the following coordinates (x, y, z) in MNI standard space: 26, −1, −20. B. fMRI activity in amygdala for each group and each condition (emotion type). Controls showed a trend toward greater activation than patients across conditions. Across groups, there was greater activation for the anger condition compared to the neutral condition. Blue solid = controls; Red dash = patients. Bars represent plus or minus the standard error of the mean. Abbreviations: ROI, region-of-interest; MNI, Montreal Neurological Institute. (For interpretation of the references to color in this figure legend, the reader is referred to the web version of this article.)

Emo Bio task: Our primary analytic approach for the Emo Bio task was to use ROIs to focus on pSTS and amygdala. The bilateral pSTS ROI was defined as outlined above and transformed to each individual's native space. Bilateral anatomical amygdala ROIs were created in individual native space based on the HarvardOxford subcortical probability atlas at a 25% probability threshold (Fig. 3A). First, separate 2 (Group) × 2 (Condition: all emotions vs. neutral) rmANOVAs were conducted on parameter estimates (i.e., beta values) for each ROI to compare within and between groups on fMRI activity during the task. Follow-up 2 (Group) x 3 (Condition: separate emotions) rmANOVAs were conducted for each ROI to explore ROI activity by emotion. A two-tailed significance threshold of *p* < .05 was applied, with additional weight given to any findings with an effect size of medium or greater.

A secondary voxel-wise whole-brain analysis was conducted using FSL. Within- and between-group averages for contrasts of interest were compared using mixed effects analysis via the FLAME (FMRIB's Local Analysis of Mixed Effects) stage 1 module (Beckmann et al., 2003). Variance estimates were calculated for each group separately. Clusters exceeding a height threshold of Z > 2.3 and a cluster probability of p < .05, corrected for whole-brain multiple comparisons are reported (Eklund et al., 2016; Friston et al., 1994).

We examined functional connectivity during the emotion conditions (excluding the neutral condition) between pSTS as a seed region and amygdala, and between pSTS and the rest of the brain. Individual subject time courses from the Emo Bio task were extracted from the preprocessed data for each subject's pSTS ROI. The time course was then entered into individual subject general linear models in FSL as a psychophysiological interaction with the combined emotion conditions. The neutral condition was entered as a separate regressor to account for variance associated with that condition. For the whole-brain voxel-wise connectivity analysis, within and between groups averages were compared using mixed effects analysis via FLAME stage 1 module using the same significance thresholds as the whole-brain GLM analysis.

## 3 Results

### 3.1 Demographic and clinical data

Table 1 provides group demographic information and clinical data for patients. There were no differences between groups in terms of age, sex, ethnicity, or parental education. Patients were chronically ill and exhibited mild to moderate symptoms. Most patients were receiving atypical antipsychotic medication; two patients were not receiving any antipsychotic medication and two patients were receiving a combination of atypical and typical antipsychotics. Mean daily doses reported in Table 1 were calculated in chlorpromazine equivalents (Andreasen et al., 2010).

**Table 1:** Demographic, clinical, and behavioral data.

	Patients *n* = 20	Controls *n* = 16	Statistic	df	*p* value	Effect size (Cohen's *d*)
Gender	7F 13M	5F 11M	ᵪ^2^ = 0.06	1	0.81	
Ethnicity/Race						
% Hispanic	10.0	12.5				
% African American	35.0	25.0				
	**Mean (SD)**	**Mean (SD)**				
Age	48.3 (9.9)	44.69 (11.1)	*F* = 1.1	1,34	0.31	
Personal education (years)	13.5 (2.9)	15.5 (1.5)	*F* = 6.2	1,34	0.02	
Parental education (years)^a^	14.5 (1.9)	15.3 (2.6)	*F* = 1.1	1,31	0.30	
Medication dosage (chlorpromazine equivalent in mg/day)	340.6 (229.5)	n/a				
BPRS						
Total score	45.1 (13.8)	n/a				
Average positive symptoms	1.04 (0.5)	n/a				
SANS						
Total score (four global scales)	39.9 (12.8)	n/a				
RFS						
Total score	15.2 (4.1)	n/a				

Emotion in biological motion task						
% Accuracy	58.3 (16.6)	65.0 (12.6)	*t* = −1.3	34	0.19	0.45
Response time (*sec*)	1.46 (0.57)	1.27 (0.42)	*t* = 1.1	34	0.28	0.38

a Data available for 17 patients, 16 controls; Abbreviations: SD, standard deviation; BPRS, Brief Psychiatric Rating Scale; SANS, Scale for the Assessment of Negative Symptoms; RFS, Role Functioning Scale.

### 3.2 Behavioral data

Performance of the 1-back task during the Basic Bio run was examined as a validity check. There were no participants in either group who failed to complete button presses periodically throughout the task. For the Emo Bio task there was no difference in accuracy or response time, collapsing across emotion conditions, between groups (Table 1).

### 3.3 fMRI data

Localizer task: Both patients and controls showed BOLD activation in bilateral pSTS, extending into medial temporal gyrus, supramarginal gyrus, angular gyrus and lateral occipital lobe.

Emo Bio task: Results of ROI analyses for the Emotion in Biological Motion Task are displayed in Figs. 2B and 3B. For the pSTS, there was a significant main effect of group (F(1, 34) = 5.83, *p* *=* .02, η_p_^2^ = 0.15) indicating greater activation in controls than patients across conditions. There was also a significant main effect of condition (F(1, 34) = 86.24, *p* < .001, η_p_^2^ = 0.72), driven by significantly greater activation for the emotion conditions (combined) compared to the neutral condition across groups. There was no significant group by condition interaction (F(1, 34) = 2.25, *p* = .14, η_p_^2^ = 0.06). Follow-up examination of ROI activity by emotion type revealed a significant main effect of group (F(1, 34) = 4.80, *p* = .04, η_p_^2^ = 0.12) indicating greater activation in controls than patients across emotion conditions. There was also a significant main effect of emotion (F(2, 68) = 6.35, *p* = .003, η_p_^2^ = 0.16), driven by significantly greater activation for the anger condition compared to the fear (*p* = .004) and happy (*p* = .07) conditions across groups. There was no significant group by condition interaction (F(1, 34) = 1.78, *p* = .19, η_p_^2^ = 0.05).

For the amygdala, there was a marginally significant main effect of group (F(1, 34) = 3.72, *p* = .06, η_p_^2^ = 0.10) indicating greater activation in controls than patients across conditions. There was a significant main effect of condition (F(1, 34) = 16.23, *p* < .001, η_p_^2^ = 0.32), driven by greater activation for the emotion conditions compared to the neutral condition (*p* < .001) across groups. There was no significant group by condition interaction (F(1, 34) = 0.80, *p* = .38, η_p_^2^ = 0.02). Follow-up examination of ROI activity by emotion type revealed no significant main effect of group (F(1, 34) = 2.45, *p* = .13, η_p_^2^ = 0.07) or emotion (F(2, 68) = 1.98, *p* = .15, η_p_^2^ = 0.06) and no significant group by condition interaction (F(2, 68) = 0.10, *p* = .90, η_p_^2^ = 0.003).

Results of exploratory whole brain analyses for the three emotion conditions combined versus baseline are detailed in Supplemental Materials (Supplementary Table 2 and Supplementary Fig. 1). Across emotion conditions, controls showed greater BOLD activity compared to patients in posterior cingulate/precuneus and bilateral fusiform gyrus. Patients did not show any areas with greater BOLD activity compared to controls.

**Fig. 4:**
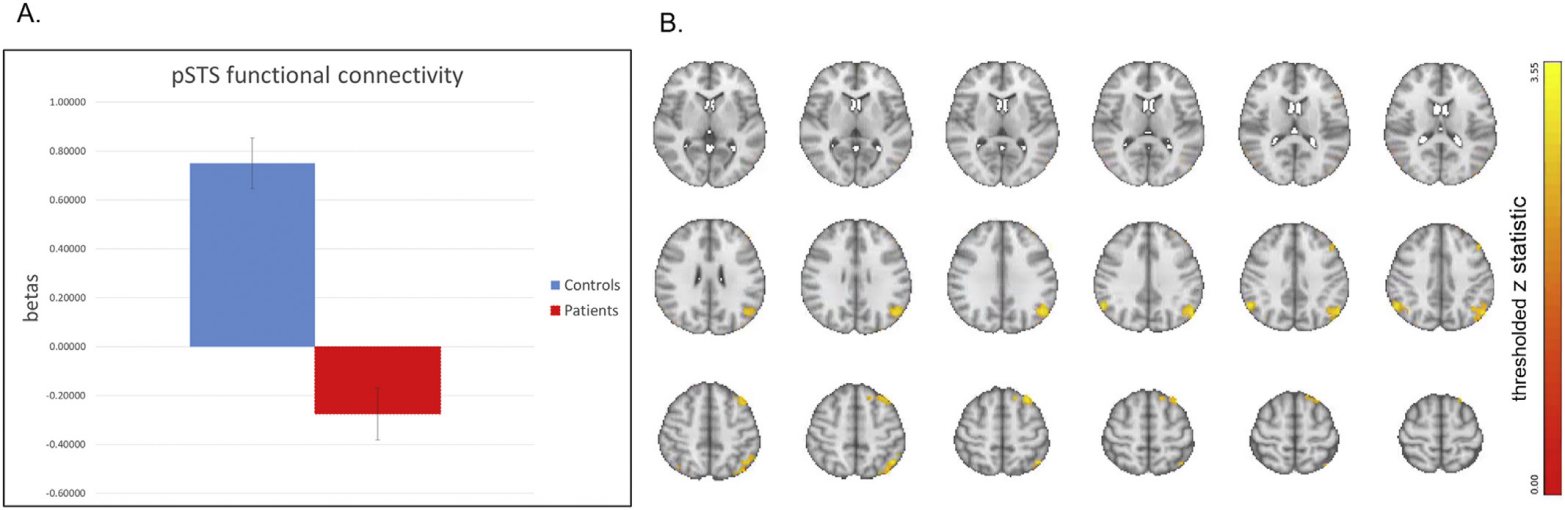
Seed-based functional connectivity between-group comparison. Controls showed significantly greater positive functional connectivity compared to patients with schizophrenia. Seed region: pSTS. Threshold: Z > 2.3, *p* < .05, corrected. For bar graphs, Blue = controls; Red = patients. Bars represent plus or minus the standard error of the mean. Axial images are radiologically oriented (left is right). A. Beta values extracted from a mask of the regions showing significant between group differences in functional connectivity. B. Axial slices of functional connectivity activation maps showing significant between group differences. See Supplementary Table 3 for coordinates of within and between group effects. (For interpretation of the references to color in this figure legend, the reader is referred to the web version of this article.)

For the connectivity analyses, we initially examined functional connectivity between bilateral pSTS as a seed and amygdala. No within or between group effects survived correction for multiple comparisons. Results of seed-based pSTS whole brain functional connectivity analyses are displayed in Fig. 4 (see Supplementary Table 3 for coordinates). Controls showed significant positive functional connectivity during the emotion conditions between pSTS and two areas: left frontal gyrus (superior and middle) and bilateral angular gyrus (extending into lateral occipital lobe). Patients did not show any areas of significant positive functional connectivity with pSTS. Direct between-group contrasts revealed significantly greater positive connectivity in controls versus patients in the same frontal and parieto-occipital regions described above. In addition, controls showed negative functional connectivity between bilateral pSTS and right anterior insula. Patients showed negative functional connectivity between bilateral pSTS and occipital lobe regions. There were no regions of significant difference between groups for negative functional connectivity.

In post hoc analyses we explored the relationship between ROI activity during the emotion conditions and measures of real world functioning (RFS total scores) and negative symptoms (SANS subtotal) in patients. Non-parametric analyses (Spearman correlations) were chosen to account for non-Gaussian distributions of the data. After Bonferroni correction for multiple comparisons, only one observed correlation was significant. During emotion conditions, patients showed a significant positive correlation between BOLD activity in amygdala and RFS total score (*r* = 0.56, *p* = .01).

## 4 Discussion

This study is the first to examine fMRI findings from a basic biological motion task with a more complex task of emotion in biological motion in schizophrenia. Results from the basic biological motion task were used to localize activity in pSTS in each participant. During the Emo Bio task, patients showed overall reduced activation compared to controls in pSTS and at a trend level in amygdala across emotions, despite similar behavioral task performance and despite comparable levels of activation in the pSTS localizer task. Further, the controls but not patients showed functional connectivity between pSTS and left frontal regions as well as bilateral angular gyrus during the Emo Bio task.

The finding of comparable activation of pSTS in schizophrenia patients and control participants during basic biological motion perception is consistent with two prior functional neuroimaging studies which used a similar task (Hashimoto et al., 2014; Kim et al., 2011). The task demands are low as participants are only required to passively view the point-light animations and respond to any repeated pattern. Thus, this task may reflect the automatic, low-level visual mechanisms involved in perception of biological motion (Giese and Poggio, 2003; Thompson and Parasuraman, 2012; Thornton and Vuong, 2004). These automatic mechanisms appear to be intact in patients.

On the other hand, it appears that any alterations in pSTS activity in patients may occur when the task is more complex. For example, during a more complex biological motion discrimination task, Kim et al. (Kim et al., 2011) found an abnormal pattern of activation in pSTS in patients. Similarly, in the current study, abnormal activation in pSTS was found only in the more complex Emo Bio task. During this task, activation in both pSTS and amygdala was reduced in patients relative to controls, despite comparable performance between the two groups. It is possible that the fMRI differences reflect increase task complexity – both in terms of the number of possible responses and that the task required both motion and emotion processing (Thompson and Parasuraman, 2012).

Analysis of functional connectivity during the Emo Bio task revealed that controls showed significant positive connectivity between pSTS and regions of dorsolateral prefrontal cortex, including superior and middle frontal gyrus, whereas patients did not. The recruitment of frontal regions during this task is consistent with the notion that additional processes may have been required for efficient performance of this task (Blake and Shiffrar, 2007).

The pattern of activation in pSTS and amygdala across conditions is also noteworthy. In both regions, activity for the emotion conditions was greater than activity for the neutral condition (though blunted overall in schizophrenia). Previous studies have found that patients with schizophrenia tend to over-attribute negative emotions (e.g., anger, fear) to neutral faces (e.g., (Pinkham et al., 2011) and to overactivate amygdala in response to neutral faces (e.g., (Hall et al., 2008)). However, in the current study patients did not show increased activation in either pSTS or amygdala in response to the neutral videos relative to other emotion types. Also, schizophrenia patients rated neutral animations similarly to controls. The differences between our findings and those from faces might be due to differences in stimulus type (e.g., static faces versus whole-body point-light animations) or to differences in difficulty level between the methods used. Further, exploration of activity by emotion type showed a significant condition effect in pSTS, but not amygdala, that was driven by greater activation during the anger condition compared to other emotion types. This heightened response to anger is consistent with the notion that threat-related signals are subject to enhanced neural processing, perhaps because they are associated with greater salience or arousal (e.g., (Fitzgerald et al., 2006; Hariri et al., 2002). The fact that we saw this effect in pSTS but not in amygdala could be attributed to reduced power to detect the effect with the current sample size.

We did not find evidence of significantly impaired behavioral performance on the Emo Bio task in our sample of patients. This was somewhat surprising, given the consistent pattern of impaired performance in prior studies of schizophrenia using similar paradigms (Okruszek and Pilecka, 2017). Still, the effect size of the difference between the groups in the current study was *d* = 0.46, which is comparable to the effect size noted in the recent meta-analysis (Okruszek and Pilecka, 2017).

The current study has some limitations. Our sample size was relatively small, which may have reduced our power to detect small task-related effects. Our schizophrenia sample was comprised of chronic outpatients, all but two of whom were taking antipsychotic medications. We do not know whether similar patterns of regional activation or functional connectivity differences would be observed in recent-onset or unmedicated individuals. In addition, we chose to utilize a basic biological motion task that did not require participants to actively discriminate biological from non-biological motion. This prevented us from being able to examine relationships between BOLD activation patterns and behavioral performance. In addition, the Emo Bio task we used included only four response options (“happy,” “afraid,” “angry,” and “neutral”), whereas previous versions of the task included a fifth emotion condition: “sad.” This modification was necessary because we were limited to a 4-button response box in the fMRI scanner, and, as a result, needed to limit the conditions to 4 emotions. We considered it to be important to include a neutral condition, a positive emotion (happy), and two versions of negative emotions (fear and anger) that are both known to activate the amygdala robustly. That meant that the sad condition was not part of our paradigm. We believe that this modification, while not ideal, had minimal impact on the results and conclusions from this study because we were still able to assess neutral, positive, and negative emotions. Finally, the current study focused on two brain regions consistently implicated in biological motion and social cue perception. Follow-up studies could examine disturbances in more regions involved in this network, as well as the network properties of these regions using graph theoretical analyses.

Social perceptual deficits in schizophrenia are well established (e.g., (Bigelow et al., 2006; Kelemen et al., 2005). The current study adds to a growing body of literature identifying the neural correlates of these deficits. Our findings indicate that the neural response is blunted when patients are required to extract more complex social information from biological motion, despite the fact that the neural correlates of basic (non-emotional) biological motion perception appear intact in schizophrenia. In addition, functional connectivity between key regions was found in controls, but not present in patients. These aberrant responses may thus contribute to social perceptual deficits in this disorder, with downstream negative effects on these individuals' ability to effectively communicate and interact with others.

## Declaration of interest

Dr. Green has been a consultant for ACADIA, DSP, Takeda, and Lundbeck, a member of the Scientific Board of Luc, and has received research funds from Forum. The rest of the authors report no biomedical financial interests or potential conflicts of interest.

## Appendix A Supplementary data

Supplemental materials Image 1
