# Supplementary material for: Aberrant patterns of neural activity when perceiving emotion from biological motion in schizophrenia
Source: Neuroimage Clin. 2018 Aug 9;20:380–7. doi: 10.1016/j.nicl.2018.08.014 (PMC6095949; doi:10.1016/j.nicl.2018.08.014)

**Supplementary Table 1.** Scanner site comparison

|  | Brain Mapping Center, UCLA | Center for Cognitive Neuroscience, UCLA | Statistics |
| --- | --- | --- | --- |
| Sample | 9 Pts 6 Cont | 11 Pts 10 Cont | ᵪ^2^(1)=.21, *p*=.65 |
| Quality control metrics |  |  |  |
| Temporal signal-to-noise ratio* |  |  |  |
| Basic Biological Motion | 40.6 (11.1) | 39.7 (11.8) | t(34)=0.24, *p*=.81 |
| Emotion in Biological Motion | 37.1 (11.5) | 34.7 (12.5) | t(33)=0.56, *p*=.58 |
| Demographics |  |  |  |
| Gender | 6F 9M | 6F 15M | ᵪ^2^(1)=.51, *p*=.47 |
| Age | 46.3 (11.6) | 47.0 (9.9) | *F(*1,34)=0.04, *p*=.84 |
| Subject education | 15.2 (2.1) | 13.8 (2.8) | *F(*1,34)=2.69, *p*=.11 |
| Parental education | 15.3 (2.2) | 14.6 (2.3) | *F(*1,31)=0.76, *p*=.39 |

*Temporal signal-to-noise ratio calculated on pre-processed data from each participant.

**Supplementary Table 2.** Results of voxel-wise analyses for emotion conditions combined (excluding the neutral condition). Maximum z-score and MNI coordinates are listed for significant within and between group effects.

| Region | P Value | Voxels | Max Z-Score | Max X (mm) | Max Y (mm) | Max Z (mm) |
| --- | --- | --- | --- | --- | --- | --- |
| *Contrast: emotion conditions vs baseline* | | | | | | |
| Controls |  |  |  |  |  |  |
| Right inferior lateral occipital cortex, occipital pole | 0.00E+00 | 39621 | 5.98 | 32 | -86 | 4 |
| Right inferior and middle frontal gyri, precentral gyrus | 1.79E-22 | 6045 | 5.09 | 48 | 12 | 28 |
| Right temporal pole, insula, frontal orbital cortex | 0.0034 | 497 | 4.02 | 24 | -2 | -12 |
| Patients |  |  |  |  |  |  |
| Left inferior and superior lateral occipital cortex, occipital pole | 2.38E-34 | 11100 | 6.25 | -28 | -90 | 18 |
| Right inferior and superior lateral occipital cortex, occipital fusiform gyrus | 1.36E-30 | 9393 | 5.73 | 50 | -72 | 12 |
| Right inferior, middle, and superior frontal gyri, precentral gyrus | 4.38E-11 | 2273 | 4.37 | 50 | 12 | 28 |
| Left paracingulate gyrus, superior frontal gyrus, anterior cingulate, supplementary motor cortex | 5.19E-10 | 1984 | 5.33 | -8 | 12 | 52 |
| Controls > Patients |  |  |  |  |  |  |
| Right posterior cingulate, precuneus cortex, lingual gyrus | 0.00091 | 595 | 3.28 | 20 | -32 | 6 |
| Left temporal occipital fusiform gyrus, lingual gyrus | 0.00265 | 515 | 3.8 | -30 | -52 | -20 |
| Right lingual gyrus, occipital pole, temporal occipital fusiform gyrus | 0.0372 | 334 | 3.17 | 2 | -80 | -6 |
| Patients > Controls |  |  |  |  |  |  |
| None |  |  |  |  |  |  |

**Supplementary Table 3.** Results of functional connectivity analyses. Regions listed showed significant functional connectivity with the posterior STS seed region during the emotion conditions (excluding the neutral condition). Maximum z-score and MNI coordinates are listed for significant within and between group effects.

| Region | P Value | Voxels | Max Z-Score | Max X (mm) | Max Y (mm) | Max Z (mm) |
| --- | --- | --- | --- | --- | --- | --- |
| POSITIVE CONNECTIVITY |  |  |  |  |  |  |
| Controls |  |  |  |  |  |  |
| Left middle frontal gyrus | 1.86E-08 | 1038 | 3.6 | -36 | 24 | 52 |
| Left superior lateral occipital cortex, angular gyrus | 2.90E-05 | 582 | 3.47 | -48 | -64 | 30 |
| Right superior lateral occipital cortex, angular gyrus | 0.0412 | 227 | 3.19 | 54 | -58 | 44 |
| Patients |  |  |  |  |  |  |
| None |  |  |  |  |  |  |
| Controls > Patients |  |  |  |  |  |  |
| Left superior lateral occipital cortex, angular gyrus | 8.34E-07 | 791 | 3.44 | -50 | -64 | 42 |
| Left middle frontal gyrus, superior frontal gyrus | 0.00166 | 371 | 3.51 | -30 | 28 | 52 |
| Right angular gyrus, superior lateral occipital cortex | 0.0403 | 228 | 3.36 | 56 | -58 | 36 |
| Patients > Controls |  |  |  |  |  |  |
| None |  |  |  |  |  |  |
| NEGATIVE CONNECTIVITY |  |  |  |  |  |  |
| Controls |  |  |  |  |  |  |
| Right anterior insula, frontal operculum | 0.0203 | 257 | 3.35 | 36 | 20 | 4 |
| Patients |  |  |  |  |  |  |
| Left superior parietal lobule | 6.56E-07 | 807 | 3.44 | -32 | -50 | 62 |
| Controls > Patients |  |  |  |  |  |  |
| None |  |  |  |  |  |  |
| Patients > Controls |  |  |  |  |  |  |
| None |  |  |  |  |  |  |

**Supplementary Figure 1. Voxel-wise whole-brain analyses.** Axial images showing significant within and between group results of voxel-wise analyses for emotion conditions combined (excluding the neutral condition). Threshold: Z > 2.3, *p* < .05, corrected. All images are radiologically oriented (left is right).

1. Controls
2. Patients
3. Controls > Patients


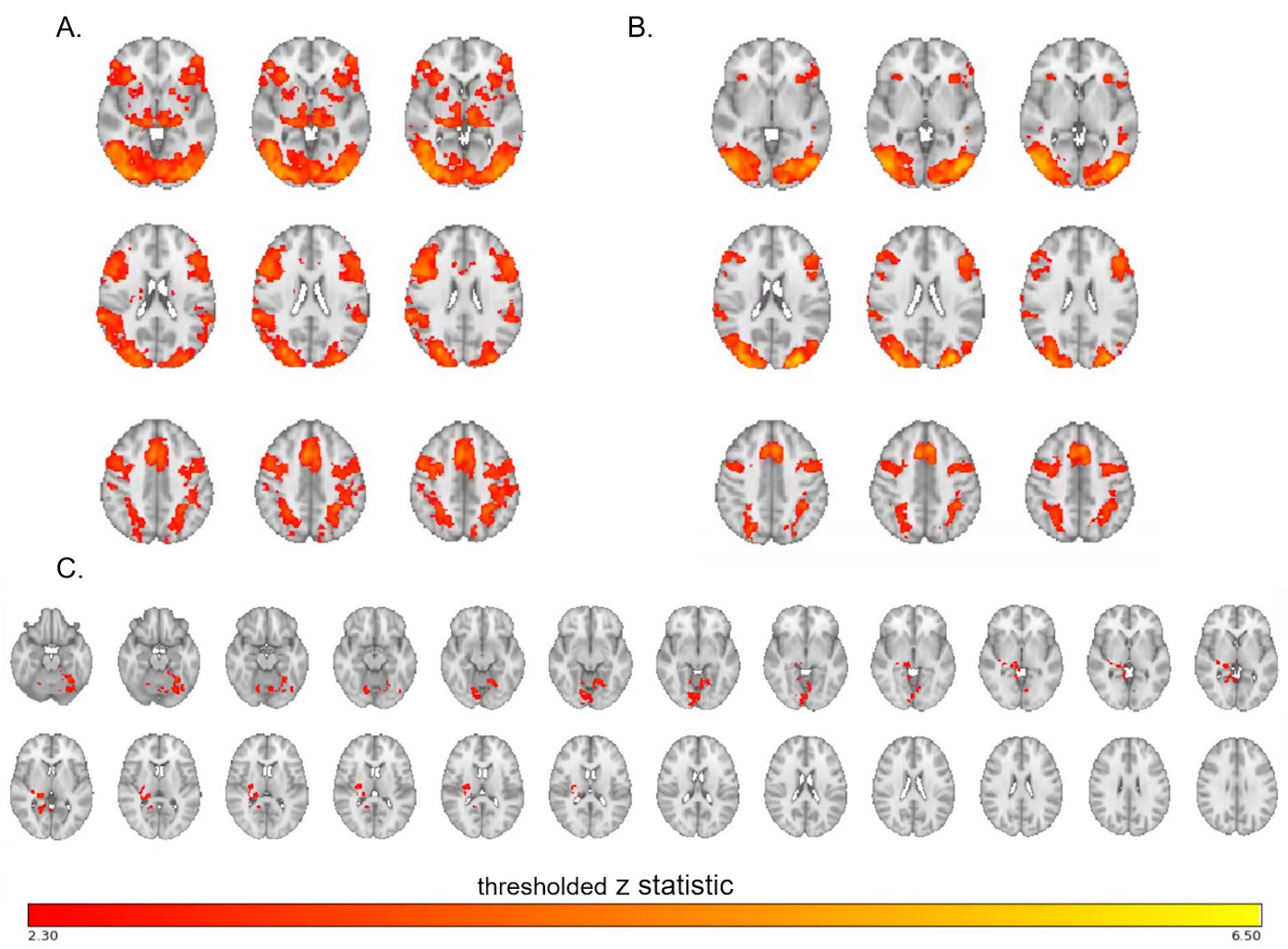

Supplement: Supplementary file 1 — Supplemental materials [file mmc1.docx]
